# Genome wide transcriptome profiling of *Fusarium oxysporum* f sp. *ciceris* conidial germination reveals new insights into infection-related genes

**DOI:** 10.1038/srep37353

**Published:** 2016-11-17

**Authors:** Mamta Sharma, Anindita Sengupta, Raju Ghosh, Gaurav Agarwal, Avijit Tarafdar, A Nagavardhini, Suresh Pande, Rajeev K Varshney

**Affiliations:** 1 International Crops Research Institute for the Semi-Arid Tropics (ICRISAT), Patancheru, Hyderabad, Telangana, 502324, India

## Abstract

Vascular wilt caused by *Fusarium oxysporum* f. sp. *ciceris (Foc*) is a serious disease of chickpea (*Cicer arietinum* L.) accounting for approximately 10–15% annual crop loss. The fungus invades the plant via roots, colonizes the xylem vessels and prevents the upward translocation of water and nutrients. Infection is initiated by conidia that invade the host tissue often by penetration of intact epidermal cells. Here, we report the characterization of the transcriptome of *Foc* sequenced using Illumina Hiseq technology during its conidial germination at different time points. Genome-wide expression profiling revealed that genes linked to fungal development are transcribed in successive ways. Analysis showed that *Foc* have large sets of germination-related genes and families of genes encoding secreted effectors, cell wall/pectin-degrading enzymes, metabolism related enzymes, transporters and peptidases. We found that metabolism related enzymes are up-regulated at early time point whereas most transporters and secondary metabolites important for tissue colonization and pathogenicity are up-regulated later as evident from the qRT-PCR. The study demonstrated that early conidial germination in *Foc* is accompanied by rapid shifts in gene expression that prepare the fungus for germ tube outgrowth, host cell invasion and pathogenesis. This work lays the foundation for facilitating further research towards understanding this host-pathogen interaction.

Fusarium wilt caused by *Fusarium oxysporum* f. sp. *ciceris (Foc*), is a major constraint in improving productivity of chickpea (*Cicer arietinum* L.) worldwide and is estimated to cause 10–15% yield loss annually. The disease can result in 100% yield losses under favourable environmental conditions. *Fusarium oxysporum* is a globally ubiquitous soil-borne fungus^1^, and is one of the most important plant-pathogens of the *Fusarium* genus ranked 5^th^ out of the top 10 plant pathogens of scientific/economic importance^2^. As a consequence of the huge loss incurred by the devastating pathogen, understanding of the mechanisms of pathogenicity is a prerequisite for implementation of control strategies to restrict the pathogen from infection and therefore combat the disease.

The asexual fungus *Foc* reproduces through the production of spores called conidia which plays a key role during plant pathogenesis. *Foc* produces three types of asexual spores - macroconidia, microconidia and chlamydospore that enable it to disperse and survive^3^. In general, germination of spores is a fundamental step in fungal development leading to the conversion of a dormant cell into growing hyphae. It involves breaking of dormancy by external signals, a pre-germination phase and then the formation of germ tube that marks the establishment of polar growth^4^. However, the process of vascular infection by *Fusarium* spp. have been defined as a multistep process involving spore germination, root recognition, adhesion to the host surface and colonization, establishment of hyphal networks through vegetative hyphal fusion, differentiation of infection hyphae, penetration of the root cortex, and hyphal proliferation within the xylem vessels^3^. The germ tube of spores or the mycelium penetrates root tips directly or enters the roots through wounds or at the point of formation of lateral roots. The mycelium advances through the root cortex intercellularly and reaches the xylem vessels. While inside the vessels, the mycelium branches and produces microconidia that germinate and penetrates the vessel wall. The mycelium also advances laterally into the adjacent vessels, penetrating them through the pits. Presumably by a combination of the processes it colonizes the xylem vessels and thus reduces or prevents the flow of water and nutrients from the roots to the upper part of the plant resulting in plant wilting.

Spore germination is governed by many intercellular signalling pathways associated with highly coordinated changes in gene expression. A series of nuclear events takes place including mitosis in the fusing hypha and nuclear migration into the receptor hypha, followed by degradation of the resident nucleus. Because of the crucial role of conidia for infection, a detailed knowledge of the molecular events during the early stages of their development is of great interest not only scientifically but also for the development of new control strategies. Over the years numerous studies have been performed to understand the genetic and biochemical processes that take place in the host during the early stages of interaction by *F. oxysporum* that are fundamental for the outcome of infection^5^,^6^,^7^,^8^. Transcriptome profiling analyses during germination have been conducted for *Neurospora crassa*^9^, the saprotrophic Chytridiomycete *Blastocladiella emersonii*, and the wheat pathogen *Fusarium graminearum*^10^. These studies revealed major changes of gene expression patterns during the pre-germination swelling stage. Genes involved in replication, protein synthesis, and degradation of storage reserves were found to be activated during the early stages of the germination process.

Very limited information is available on the molecular events involved in early developmental stages of *Foc* such as conidial germination that are of key relevance during the establishment of infection. Therefore, in the present study, we performed a transcriptome analysis of the conidial germination of *Foc* in order to get insight into the molecular events during early stages of the germination process. We have used next generation sequencing to perform a comprehensive analysis of gene expression throughout conidial development. This expression analysis will provide new insights for further studies directed at understanding fungal conidiogenesis and its molecular regulation. Moreover, it will provide a better understanding of the physical factors affecting sporulation and conidial germination that would help in better understanding of the epidemiology and the control of vascular wilt diseases. Analysing the expression of plant defence related genes or fungal pathogenesis genes which have been known to be involved in such an interaction; as well as characterizing new genes for their role in the host pathogen interaction can enable the in-depth search of functions of such genes.

## Results and Discussion

### Conidial germination of *Foc*

**Figure 1:**
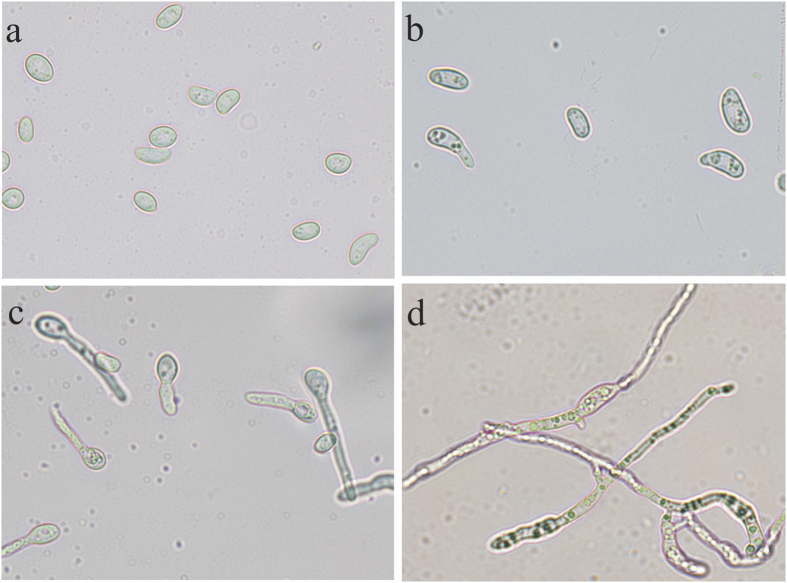
Microscopic examination of conidial germination at different time points. (**a**) 0 h- resting conidia. (**b**) 2 h-initiation of hyphal swellings (**c**) 6 h- emergence of germ tube. (**d**) 24 h- hyphal elongation and branching.

Pre-germination swelling was observed to start after about 1 h and continued until germ tube emergence. Approximately 20% of the conidia germinated after 2 h, however, it took more than 6 h when all conidia had germinated (Fig. 1). At 12 h, 100% germlings were seen and after 24 h the germlings had resumed elongation. The pattern of conidial germination was preferentially unipolar (92% unipolar), with most microconidia producing a single germ tube. Vegetative hyphal fusions between germ tubes were also observed.

### Comparative expression analysis during conidial germination

**Figure 2:**
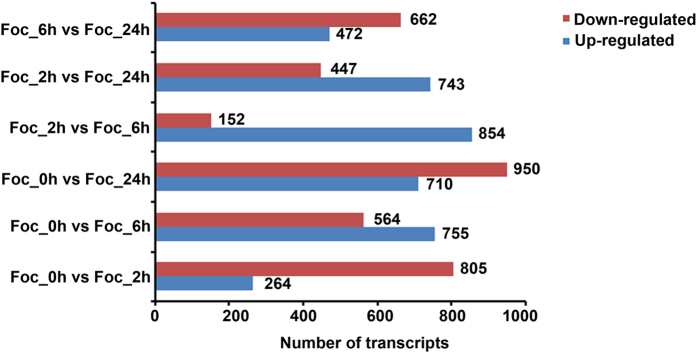
Differentially regulated transcripts with significant expression represented by log_2_fold change ≥2 and ≤2 at various time points. X-axis represents numbers of up-regulated and down-regulated transcripts, Y-axis represents six combinations of time points.

In accordance to our microscopic observations, four time points (0 h, 2 h, 6 h and 24 h) were chosen and high-throughput RNA-Seq analysis was performed. A total of 44,513,068, 67,255,054, 64,660,426 and 66,468,751 high quality raw reads were generated respectively after sequencing from all four samples and used for transcriptomic analysis across different time points. The transcripts abundance in the sample was quantified on the basis of number of sequence reads mapped on the *Fusarium oxysporum* f. sp. *Lycopersici (Fol*) reference genome, with total number of mapped reads per sample (Table 1). Total number of genes expressed in *Foc*_0 h, *Foc*_2 h, *Foc*_6 h and *Foc*_24 h was 12,679, 12,387, 12,917 and 12,888 respectively. Cufflinks analysis yielded specific data across all developmental time points that were further evaluated for stage-specific changes in global transcript abundance by performing pairwise comparisons between different time points (*Foc*_0 h vs 2 h, *Foc*_0 h vs_6 h, *Foc*_0 h vs 24 h and *Foc*_2 h vs 6 h, *Foc*_2 h vs 24 h, *Foc*_6 h vs 24 h). The distribution of both the significant as well as non-significant genes which were found to be differentially expressed has been represented in the volcano plot for all the combinations (Supplementary Figure 1). The largest changes were observed at 0 h vs 24 h (Fig. 2) when most morphological changes, including germ tube outgrowth and hyphal branching occurred, whereas, significant changes were observed at other time points according to differences in their FPKM values.

### Gene Ontology (GO) classification

**Figure 3:**
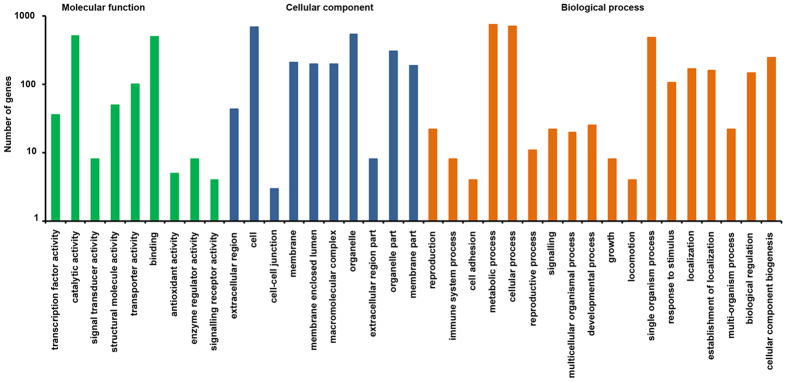
Functional classification of differentially expressed transcripts of *Foc* based on gene ontology (GO) as cellular component, molecular function and biological process.

A total of 7,378 differentially expressed sequences were obtained from which 3,798 up-regulated and 3580 down-regulated transcript were identified that showed significant up-regulation (P value ≤ 2) and down-regulated (P value ≥ 2) in six combination (Fig. 2). From the data set, 2,659 unique sequences were identified that were annotated based on BLAST results against Swiss-Prot and Tremble databases. Among these unique sequences, 1,569 were assigned to the ‘molecular function’, 1,671 to ‘biological process’ and 1,380 to ‘cellular component’ (Fig. 3). Among the 1,569 unique sequences under ‘molecular functions’ at different time points, 828 transcripts were up-regulated and 741 were down-regulated. Among up-regulated sequences 60.62% were predicted to have binding activity and 61.83% were predicted to have catalytic activity. Of the transcripts predicted to be involved in biological processes, 890 were up-regulated and 781 were down-regulated. The majority of these up-regulated transcripts (83.25%) represent ‘metabolic processes’. In ‘cellular component’, 90.18% transcripts were integral to cell part, 40.18% were integral to components of the cell organelle. Details of differentially expressed sequences under different categories are provided in Supplementary Table 1.

### Changes in gene expression pattern during conidial germination

#### Nutritional requirements during conidial germination

**Figure 4:**
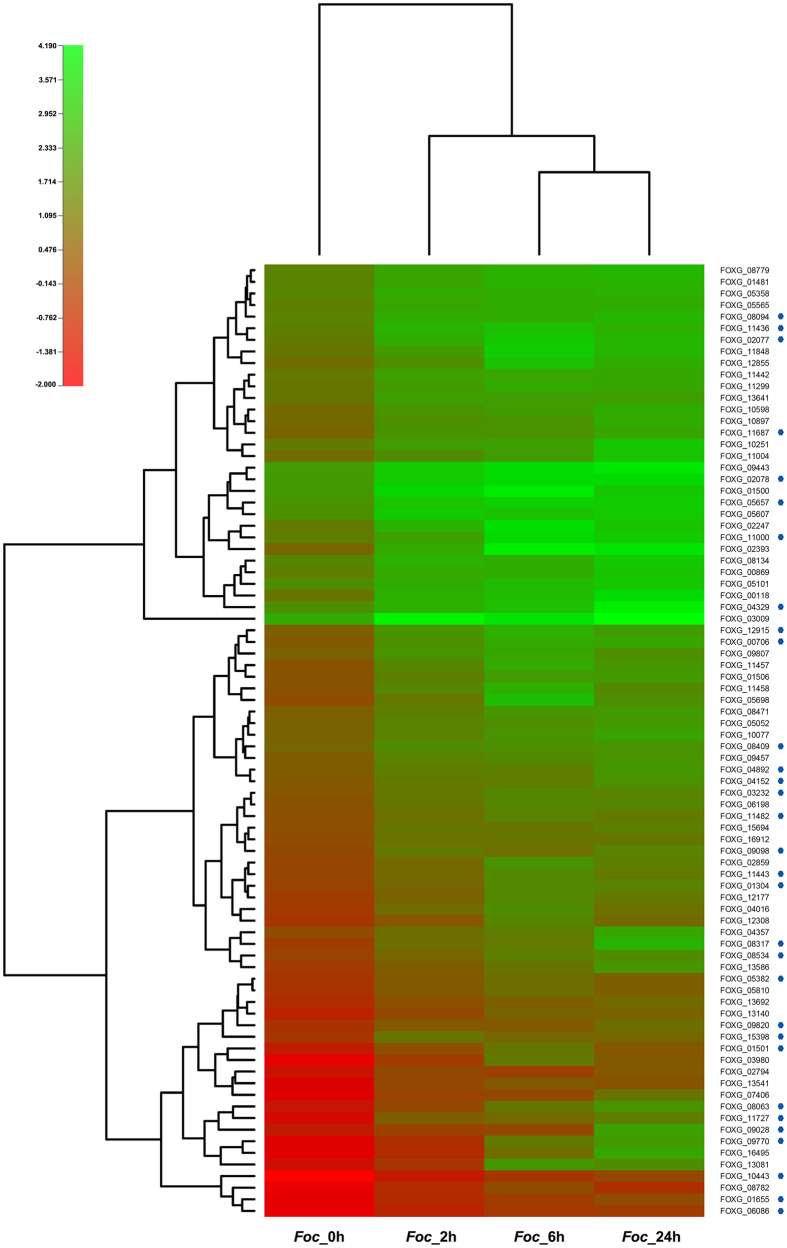
Heat map showing up-regulated transcript (log2fold ≥ 2) common in four time points. Genes with specific functions were labeled with blue dot and corresponding gene names with biological functions were listed in Table 2.

Carbohydrate synthesis such as the production of sugar, alcohols and organic acids is the most active portion of the intermediate metabolism category as they are highly expressed during germination process. Several carbohydrate metabolism genes related to oxidative phosphorylation, glycolysis, pentose phosphate pathway were found to be up-regulated during conidial germination of *Foc* (Supplementary Table 2). In most conidial germination studies, sugars have been found to be good source of carbon that oxidized more rapidly and supported good conidial germination^11^,^12^,^13^. At 0 h vs 6 h, we found expression of transcripts encoding enzymes like glycogen phosphorylase (FOXG_06319) and pyruvate carboxylase (FOXG_01733) associated with glycogen breakdown and or gluconeogenesis indicated energy utilization sources for the synthesis and expansion of hyphal growth during conidial germination. We identified many transcripts sharing similarity to genes encoding proteins involved in amino acid metabolism and protein synthesis (Fig. 4). During 0 h vs 2 h, 0 h vs 6 h and 0 h vs 24 h, expression of arginase (FOXG_12915) was found to be commonly up-regulated (Fig. 4), however, expression was more prominent at early hour (0 h vs 6 h), supporting the observation of Turner and Weiss, (2006). They examined developmental expression of the two arginase transcripts and proteins during conidial germination in *N. crassa* and found storage of both arginase proteins in conidia and temporal expression of *aga* transcripts during early germination^14^. Expression patterns of these genes when validated through qRT-PCR was found to be almost similar. For instance, significant up-regulation of glycogen phosphorylase and pyruvate carboxylase gene was found at 6 h, whereas arginase transcripts slightly down-regulated at similar time point (Supplementary Table 5).

Fatty acids are essential for fungal spore formation as they form spore membranes and lipid in stored form serve as an energy reserve to be utilized by the fungus during subsequent germination^15^. We also found several genes encoding putative enzymes related to fatty acid biosynthesis induced during germination (Supplementary Table 2). *Fusarium* macroconidia and especially chlamydospores are known for having high lipid content that appears to be utilized during endogenous respiration by ungerminated macroconidia^16^,^17^. We found that Enoyl coA hydratase (FOXG_09784) one of the primary enzyme of β-oxidation and malate synthase (FOXG_03099) one of the most important enzymes for glyoxylate cycle showed up-regulation at different time points and confirmed with qRT-PCR (Supplementary Table 5). Fatty acid metabolism and glyoxylate cycle is critical to a range of other biochemical pathways associated with fungal developmental process^18^,^19^.

#### Cellular transporters involved in conidial germination

The transcriptional analysis indicated that the genes for cellular transport (including inorganic ion transport) were among the significantly up-regulated genes at various time points. Different transporters are essential for import of the nutrients and export of secondary metabolites and other toxic compounds. Analysis indicated significant up-regulation of Zn ion transporter (FOXG_05915) during 6 h vs 24 h (log_2_fold change 5.28). Significant increases in metal ion transporters have also been reported during conidial germination in *N. crassa*^20^,^21^. The regulation of Zn homeostasis was found to be essential for *Aspergillus fumigatus* virulence^22^. Apart from that, a marked increase in transcript involved in Major Facilitator Superfamily (MFS) type drug efflux transporter protein (FOXG_04325) had shown a significant up-regulation in 6 h vs 24 h (log_2_fold change 7.59). MFS proteins were usually involved in the transport of a wide range of substrates^23^. Similarly, several ATP-binding cassette (ABC) transporters (FOXG_11983) were preferentially expanded at 0 h vs 6 h but down-regulated afterwards (Supplementary Figure 2). Up-regulation patterns for all the transporter proteins was found to be similar when validated through qRT-PCR. Zn ion transporter regulator and ABC transporter were highly up-regulated at 2 h, whereas MFS transporter was highly up-regulated at 24 h (Supplementary Table 5).

#### Cell wall biosynthesis and membrane modification for conidial developmental process

The fungal cell wall is a complex structure composed typically of chitin, 1, 3-β- and 1, 6-β-glucan, mannan and proteins. For growing hyphae, cell wall polymer branching and cross-linking is required for the maintenance of wall plasticity during morphogenesis, that depend upon the activities of a range of hydrolytic enzymes intimately associated with the fungal cell wall^24^. In our study, expression of several cell wall modifying enzymes like glucan synthase along with several extracellular cell wall glucanase (FOXG_01953, FOXG_11947), endoglucanase-5 showed common expression at 2 h vs 6 h and 24 h (FOXG_10638). Cell wall macromolecule catabolic process (FOXG_17723), cell wall glucanosyltransferase (FOXG_08810), endochitinase (FOXG_12882) were involved in chitin catabolic process and cell wall macromolecule catabolic process (FOXG_03822) were found to be induced in hyphae (Supplementary Tables 2 and 5). Similarly, enzymes like pectin methyl esterase (PME) (FOXG_12330) associated with fungal cell wall modification and organization (FOXG_13795) were also up-regulated in a continuous manner during 0 h vs 24 h and 2 h vs 24 h. The analyzed results were validated with qRT-PCR (Supplementary Table 5). The capacity to produce PME is common among the vascular Fusaria^25^ and may be an important factor in vascular discoloration, plugging and wilting^26^. It has been reported that a virulent strain of *Fol* produced 2–3 times more PME than a less virulent strain^27^. Further, we found induced expression of chitin synthase gene which is involved in production of hyphae and fungal virulence and septation of conidia^28^,^29^ throughout the time points (log_2_fold change 4.65, 6.43 and 7.74 in 0 h vs 2 h, 0 h vs 6 h and 0 h vs 24 h respectively). These studies have revealed that chitin synthase gene is highly expressed in young conidia, and plays diverse roles in hyphal growth, conidiogenesis, appressorium development, and pathogenesis^30^,^31^.

#### Specific proteins involved in conidial germination

Expression of conidiation specific proteins were found up-regulated during late hours of development. The clock-controlled gene (*ccg*) reportedly play a role in fungal conidial development e.g. ccg-2 in *N. crassa*^32^. Inactivation of this gene results in altered conidiophore morphology and abolishes the normal circadian rhythm of asexual macroconidial development of *N. crassa*^33^. In this study, a gene similar to *ccg* (ccg-9, FOXG_13755) was expressed at a higher levels in the germ tube and mycelium than in the young conidium. It showed down-regulation at 0 h vs 6 h but up-regulation at later time points such as 2 h vs 6 h (log_2_fold change 2.7), 2 h vs 24 h (log_2_fold change 5.35) and 6 h vs 24 h (log_2_fold change 2.65). This suggested that *ccg* could play dual regulatory roles in the circadian clock and during fungal spore development. Several homologs of conidiation-related genes from different ascomycetes either involved in or differentially regulated during conidiation in *Aspergillus nidulans* and *N. crassa* was also reported to be up-regulated^34^,^35^. Some of the transcripts such as phytoene dehydrogenase (FOXG_12143), opsin (FOXG_15406), protein bli-3 (FOXG_06342) showed conidiation specific expression at 6 h vs 24 h and found to be highly up-regulated at 24 h when validated in qRT-PCR (Supplementary Table 5).

An increased level of transcripts encoding cAMP-dependent protein kinase regulatory subunit (FOXG_05517) and protein kinase domain-containing protein (FOXG_08525, FOXG_03853) was observed. It was verified that up-regulation of these genes took place after 6 h in qRT-PCR analysis (Supplementary Table 5). It has been suggested that cAMP signaling plays a critical role in the transition from aerial growth to proconidial chain formation^36^,^37^. An increased regulation of G2 specific protein kinase nim1 (FOXG_06086) was observed (Fig. 4) which is involved in protein phosphorylation and that plays an important role in mitotic regulation was observed.

#### Apoptosis inducing factor

In sexual and asexual (conidia) spore formation, apoptotic-like cell death has been associated. We found that apoptosis inducing factor (FOXG_15750) has been up-regulated in the *Foc* conidial germination process across the time points. Apoptosis associated gene, metacaspase 1 (FOXG_06044) was up-regulated during the initial time points and down-regulated at 6 h vs 24 h (Supplementary Table 2). This enzyme was reported in both *F. graminearum* and *F. verticillioides* and found to be markedly up-regulated during the middle phases of perithecium development in both species, suggesting their importance in the developmental process.

Enzymes involved in nucleotide metabolism, transferase, oxidoreductase activities were found most abundant. It would be interesting to explore their function during establishment of *Foc* disease. Transcripts involved in alcohol dehydrogenase activity (FOXG_09028) have also shown a marked increase in their expression. There was an up-regulation of gamma-glutamyl transferase and gamma-glutamyl transpeptidase, involved in gamma-aminobutyric acid catabolic process that has been reported to be important for conidiation and pathogenicity^38^.

#### Pathway triggered during conidial germination

**Figure 5:**
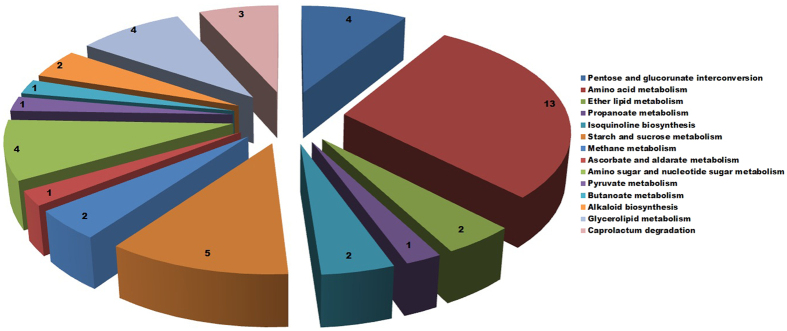
Pathways mapped with up-regulated transcripts at different combinations of time points.

**Figure 6:**
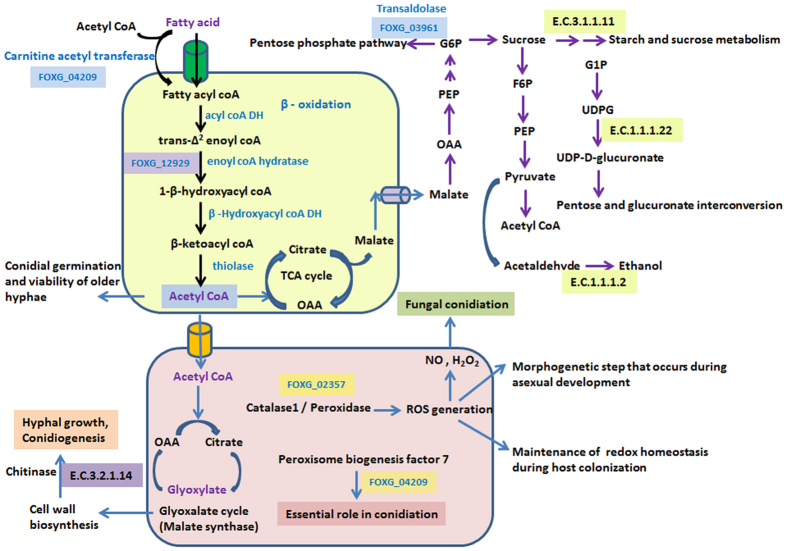
Schematic representation of major biochemical pathways up-regulated during conidial germination.

Total 334 up-regulated and 327 down-regulated transcripts (Supplementary Table 3) were mapped to 37 metabolic pathways according to significant log_2_fold values (Fig. 5). Some of these pathways were found to be common between up and down-regulated transcripts at different time period. Among the up-regulated transcripts the major pathways activated included glycolysis, gluconeogenesis, pentose and glucoronate interconversion, fatty acid biosynthesis, purine metabolism, amino acid metabolism and tetracycline biosynthesis (Fig. 6).

Dormant conidia (0 h) vs pre-germinating conidia (2 h) showed the most divergent transcript profile in comparison to other examined time points. The major pathways that were associated, especially during this stage of germination, involved starch and sucrose metabolism (EC 1.1.1.22, EC 1.1.1.10, EC 1.1.2.2, EC 3.1.1.11) (Supplementary Figure 3), amino sugar and nucleotide sugar metabolism (EC 2.4.1.16) (Supplementary Figure 4), amino acid metabolism (EC 3.5.3.1) (Supplementary Figure 5), ether lipid metabolism (EC 3.1.1.47), pyruvate metabolism (EC 3.1.2.6), propanoate and butanoate metabolism (EC 2.6.1.19, EC 1.2.1.16) and isoquinoline biosynthesis process (EC 1.4.3.21) (Supplementary Table 4). These pathways strongly indicated changes in gene expression during conidial germination that mainly focus on functions related to energy metabolism.

Cell wall degrading enzymes act upon exposed cellulosic and pectinaceous wall components by means of which vascular Fusaria obtain substrates required for vegetative growth and reproduction within invaded xylem vessels^39^,^40^. One of the major pathway found to be activated during conidial germination process was related to cell wall degradation. Several cell wall modifying enzymes (CWME) (EC 2.4.1.16, EC 3.2.1.14, EC 3.1.1.11) (Supplementary Table 4) were identified that are predicted to degrade chitin, cellulose, hemicellulose and pectin, may contribute to initial host penetration, together with a larger set of enzymes that potentially remodel the fungal cell wall. It has been reported that *F. oxysporum* produces several enzymes that act upon the pectic and cellulose components of cell walls associated with colonizing susceptible tissue by overcoming host resistance^41^.

Once the germination process initiates, the breakdown of dormancy is associated with activation of amino acid, nucleotide sugar, lipid and carbohydrate metabolisms. These pathways are related to the synthesis and expansion of hyphal and haustorial walls and membranes etc. However, during the later stages of germination, activation of fructose and mannose metabolism, methane metabolism, caprolactum degradation indicated an alternative means for replenishing energy in starved culture.[Table t2]

Bisphenol degradation pathway related to lignin degradation has also been found to be activated and (Supplementary Figure 6) reported to be essential to degrade secondary plant cell wall composed of lignin for establishment of infection. Other important pathways that found to be activated were isoquinoline alkaloid biosynthesis and drug metabolism (Supplementary Figure 7). Isoquinoline alkaloids biosynthesized from tyrosine (one of the largest groups of natural products) mainly occur in higher plants and influences macroconidia germination in Fusarium species. Enzyme related to drug metabolism - cytochrome P450 (CYP) (EC 1.14.13.8) was activated during late hours of germination (Supplementary Table 4) and has been reported to play an important role in biosynthesis of secondary metabolites (SM) that possesses a genetic connection to sporulation. Secondary metabolite production usually commences in the growth of the microbe upon entering the stationary or resting phase and often plays a role in triggering plant cell death and disease. In *A. nidulans*, a relationship between conidial germination and the antibiotic production has been described and suggested that common elements in signal transduction regulate sporulation and antibiotic metabolism. Polysaccharide synthesizing enzyme, phosphomannomutase (PMM) (EC 5.4.2.8), a key enzyme of fructose and mannose metabolism showed up-regulation at 2 h vs 24 h. PMM plays crucial role in fungal pathogenicity by protecting it against plant defense mechanisms involving chitin-hydrolysing enzymes or 1, 3-β-glucanases.

## Conclusions

*Foc* transcriptome profile is well equipped with genes encoding cell wall modifying enzymes and an array of enzymes related to secondary metabolite synthesis potentially helpful for degradation of the plant cell wall during infection process and well establishment of infection inside the host tissue, respectively. Majority of genes represented a differential expression of diverse array of enzymes indicative of the dramatic variation in metabolic and physiological changes that occur during conidial germination. Large proportion of highly expressed genes found in the energy metabolism reflects the intense sporulation occurring during the early stage. *Foc* transcriptome will be useful genomics resource for further investigating candidate genes involved in various metabolic pathways. This will further accelerate our efforts towards discovering pathogenicity mechanisms which eventually will help in improvement of Fusarium wilt disease resistance in chickpea.

## Materials and Methods

### Fungal strain and culture conditions

*Fusarium oxysporum* f. sp. *ciceris* monoconidial isolate (*Foc*-38-1) isolated from chickpea in Patancheru (Hyderabad, India) was used for this study. The isolate represents the most virulent race of this formae specialis (race 1). For conidia production, *Foc* culture was grown in potato dextrose broth (PDB) at 25 °C with shaking at 120 rpm for 4 days. The culture was harvested by filtering through mira cloth and conidial suspension was collected. The conidial suspension was washed with repeated changes of sterile distilled water in a centrifuge at 5000 rpm. For the induction of germ tubes the harvested conidia were re-suspended in germination solution (0.5 g MgSO_4_.7 H_2_O, 1 g NH_4_NO_3_, 1 g KH_2_PO_4_ & 15 g glucose in 1 liter of water) at a concentration of about 10^6^ conidia per ml and the number of spores present in 1 ml of the suspension was estimated with the help of a haemocytometer. The original spore suspension was diluted serially to get the suspensions of up to 10^−4^ dilutions. The number of spores present in 1 ml of each suspension was estimated with the help of a haemocytometer.

### Microscopy

For light microscopy study of germination patterns, Olympus CX41 (Olympus Bioscapes) upright microscope driven by Q Capture Pro 7 software (Advanced imaging concepts Inc, NJ 08540, USA) and equipped with PLCN series of plan Achromat objectives, Q imaging Micro publisher 5.0 RTV camera and a specific filter for GFP was used. Images were recorded using a Q imaging camera at 400X magnification.

A drop of 0.05 ml of conidial suspension was added in each cavity slide and covered with coverslip. The inoculated slides were then placed in big petri dishes (25 cm diameter) lined with moist filter paper to provide high humidity. The slides present in moist chambers were incubated at 25 °C with 12 hr photoperiod till conidia develop vegetative hyphae with fusion between them. Before incubation in the cavity slides, the germinated spores were examined microscopically. A spore was considered to have germinated if the germ tube was about half or more than half of the length of spore. Five replications were maintained for each dilution. The number of spores germinated in each of the dilution was estimated at the end of 0, 2, 3, 4, 5, 6, 7, 8, 9, 10, 12, 16 and 24 h of incubation periods from which 0 h, 2 h, 6 h and 24 hours time points have been selected according to the progression of germ tube emergence. The germination was recorded as 100 conidia/replication under several microscopic fields and conidial germination was calculated using the formula.

% germination = (No. of spores germinated in total number of spores counted/Total no. of spore)*100.

### RNA isolation, purification and cDNA library preparation

Total RNA was isolated from all 4 lyophilized samples by using the Xcelgen fungul RNA Mini kit according to the manufacturer’s protocol (Qiagen, Germany) from each selected time points 0 h, 2 h, 6 h and 24 hours of the *Foc* culture selected based on progression of germ tube development. These time points of conidial germinations were referred as *Foc*_0 h, *Foc*_2 h, *Foc*_6 h and *Foc*_24 h. The integrity of total RNA was assessed on 1% denaturing agarose gels and quantity was measured with a NanoDrop ND-1000 spectrophotometer (NanoDrop Technologies, USA). Total RNA samples with 28 S/18 S ratios in a range from 1.8 to 2.0 and RNA integrity index from 8.0 to 10.0 were selected for further processing. Approximately 5 μg of polyA mRNA was isolated from total RNA using oligo dT beads as per manufacturer’s instructions and pair end cDNA sequencing libraries were prepared for all 4 samples, according to the illumina TruSeq RNA library preparation kit. To avoid priming bias, the mRNA was first fragmented into small pieces using divalent cations at elevated temperature and converted to double-stranded cDNA using SuperScript II primed by random primers. The resulting cDNA was purified using the QIAquick PCR Purification Kit (Qiagen) and subjected to end-repair followed by phosphorylation. The cDNA fragments were 3′ adenylated and purified again for subsequent adapter ligation. A range of ligated cDNA samples were selected on agarose gel and purified by gel extraction kit. Fifteen cycles of PCR amplification was performed on selected samples to enrich the library and PCR products were again purified using QIAquick PCR purification Kit (Qiagen). cDNA quantification and qualification was performed on Caliper LabChip GX using HT DNA High Sensitivity Assay Kit and the cDNA libraries for each time point were sequenced using the Illumina Genome Analyzer. The libraries of all samples came into size range of 290–303 bp. Two biological replicates of every sample were analyzed. The raw Illumina sequencing data were deposited in GEO (http://www.ncbi.nlm.nih.gov/geo/) at NCBI.

### Analysis of RNA sequences

Paired end sequencing allows the template fragments to be sequenced in both the forward and reverse directions. Cluster generation was carried out by hybridization of template DNA molecules onto the oligonucleotide-coated surface of the flow cell. Immobilized DNA template copies were amplified by bridge amplification to generate clonal DNA clusters. This process of cluster generation was performed on cBOT using TruSeq PE Cluster kit v3-cBot-HS. The kit reagents were designed to allow selective cleavage of the forward DNA strand after resynthesis of the reverse strand during sequencing. The copied reverse strand was then used to sequence from the opposite end of the fragment. TruSeq SBS v3-HS kit was used to sequence DNA of each cluster on a flow cell using sequencing by synthesis chemistry on the HiSeq2000. Approximately, 15 million PE reads were generated per sample.

### RNA-seq reads mapping and differential gene expression analysis

The reference genome of *Fusarium oxysporum* f. sp *lycopersici* was downloaded from the given link: http://www.broadinstitute.org/annotation/genome/fusarium_group/MultiDownloads.html (*F. oxysporum* 4287). Raw reads were filtered using Trimmomatica tool to obtain high-quality reads by removing low-quality reads with Q < 25. After trimming low-quality bases (Q < 25) and adaptor sequences, the resulting high-quality reads were aligned onto the downloaded reference genome. In order to perform spliced alignment of the reads against the reference genome Tophat v1.3.3^42^ and Bowtie v0.12.9 software^43^ was run with default settings. The reads were assembled, normalized and gene expression level was quantified in terms of fragments per kilobase of exon per million fragments mapped (FPKM) using Cufflinks and Cuffdiff v1.3.0^44^. Heat map were generated using FPKM values by using heatmap2 function of R package (http://www.R.project.org/.). Pearson correlation was performed for all four time points and Pearson correlation matrix was generated by using excel Pearson function.

Differential gene expression was carried out for genes expressed in all the combinations of time points i.e. *Foc*_0 h vs *Foc*_2 h, *Foc*_0 h vs *Foc*_6 h, *Foc*_0 h vs *Foc*_24 h, *Foc*_2 h vs *Foc*_6 h, *Foc*_2 h vs *Foc*_24 h, *Foc*_6 h vs *Foc*_24 h samples respectively. Cuffdiff followed by cufflinks and cuffmerge was used to identify DE genes based on false discovery rate (FDR) adjusted p-values, after Benjamini-Hochberg correction for multiple testing^45^ and log_2_fold change values of > = +2 and < = −2 (excluding the genes with infinite expression) with significance marked as ‘yes’.

### Gene ontology and KEGG pathway enrichment analyses

Cytoscape (version 3.1.0)^46^ and plugin BiNGO^47^ were used for GO and KEGG^48^ pathway analyses. Overrepresented GO terms were identified by using hypergeometric test with a significance level of 0.05 and Benjamini-Hochberg method with false discovery rate (FDR) correction of the p-values. The GO Slim terms for molecular function, biological process, and cellular component categories associated with the best blastx hit of the *Saccharomyces cerevisiae* protein were assigned to the corresponding Fusarium transcript. For pathway analysis of the differentially expressed genes (DEG’s) compared at various time points, the best DEG’s based on the respective log_2_ threshold values (minimum of 3 for up-regulated and −5.5 for down-regulated genes) at different time points were obtained. The DEGs thus obtained were then searched against NCBI-nr protein database using blastp program embeded in Blast2GO software^49^ with an E-value threshold of less than equal to 1e-15. Proteins for which the enzyme ids were obtained were represented in respective pathways.

### qRT-PCR analysis

The time dependent expression patterns of some selected genes involved in conidial germination was validated with qRT-PCR analysis. Following total RNA isolation from all 4 lyophilised samples using Nucleospin RNA isolation kit (MACHEREY-NAGEL GmbH & Co. KG, Germany) complementary DNA synthesis was performed using SuperscriptIII reverse transcriptase (Invitrogen, USA). qRT-PCR was performed using KAPA SYBR® FAST qPCR Kit (KAPA Biosystem, Wilmington, Massachusetts) on a Mastercycler® RealPlex system (Eppendorf, Hamburg, USA). Thermo-cycling and melt-curve conditions were as described by James *et al.*^50^. For each sample, three biological replicates were used. The averaged Ct values in three replicates of analysed genes were normalized with the Ct values of the internal control 18 S gene, and standard deviations and errors were calculated. Absolute gene expression levels relative to Foc 18 S were calculated by the 2^−ΔΔCT^ method where Ct is the cycle threshold value^51^. Primer sequences are listed in Supplementary Table 5.

## Additional Information

**How to cite this article**: Sharma, M. *et al.* Genome wide transcriptome profiling of *Fusarium oxysporum* f sp. *ciceris* conidial germination reveals new insights into infection-related genes. *Sci. Rep.* **6**, 37353; doi: 10.1038/srep37353 (2016).

**Publisher’s note:** Springer Nature remains neutral with regard to jurisdictional claims in published maps and institutional affiliations.

## Supplementary Material

Supplementary Information

## Figures and Tables

**Table 1:** Summary of sequence reads generated by RNA-Seq analysis.

Samples	Raw reads	Filtered reads	Total mapped reads	Uniquely Mapped reads
*Foc*_0 h	46269281	44513068	35684403	32645201
*Foc*_2 h	69895094	67255054	52259371	47597129
*Foc*_6 h	68489414	64660426	52621909	46663478
*Foc*_24 h	70126052	66468751	55143238	48966161

**Table 2 t2:** Up-regulated transcripts with significant expression.

NAME	Putative biological function	*Foc*_0 h	*Foc*_2 h	*Foc*_6 h	*Foc*_24 h
FOXG_05358	Reduced viability upon starvation protein 167	35.51	168.06	193.62	216.19
FOXG_05565	Fimbrin	28.75	140.34	198.27	197.66
FOXG_08094	Succinyl-CoA:3-ketoacid-coenzyme A transferase	31.12	204.35	185.99	308.43
FOXG_11436	Succinate-semialdehyde dehydrogenase	26.74	283.76	501.63	231.36
FOXG_02077	Succinate-semialdehyde dehydrogenase	20.41	202.5	608.89	291.53
FOXG_11687	FMN-dependent dehydrogenase	8.04	63.96	62.37	132.91
FOXG_02078	4-aminobutyrate aminotransferase	86.31	693.01	1494.98	1450.29
FOXG_05657	L-xylulose reductase	62.87	509.82	996.83	783
FOXG_11000	Flotillin domain-containing protein	25.06	114.43	1488.56	754.9
FOXG_04329	Phosphatidylserine decarboxylase	51.57	230.19	478.12	3399.18
FOXG_12915	Arginase	6.47	62.45	228.23	101.59
FOXG_00706	AhpC/TSA family protein	4.76	54.44	184.71	147.52
FOXG_08409	Acyl-CoA dehydrogenase	8.71	48.07	53.28	62.2
FOXG_04892	Vacuolar protein sorting-associated protein vps13	5.47	26.06	25.73	78.5
FOXG_04152	Bifunctional P-450:NADPH-P450 reductase	4.76	20.99	23.45	68.23
FOXG_03232	2-nitropropane dioxygenase	4.17	20.18	38.11	29.97
FOXG_11482	Repressible alkaline phosphatase precursor	3.15	12.71	30.43	19.9
FOXG_09098	General stress protein 39	2.33	21.51	12.87	29.69
FOXG_11443	Aldehyde dehydrogenase, mitochondrial precursor	2.09	10.7	41.12	21.2
FOXG_01304	2-methylcitrate synthase, mitochondrial precursor	1.77	8.7	39.36	31.1
FOXG_08317	Skeleton binding protein	1.27	13.46	19.08	258.61
FOXG_08534	phospholipase D PLD	1.93	9.05	26.75	50.64
FOXG_05382	3-oxoacyl-(acyl-carrier-protein) reductase	0.98	4.77	11.7	6.59
FOXG_09820	Phosphorylcholine phosphatase	0.89	4.7	5.06	13.92
FOXG_15398	Multidrug resistant protein	1.05	18.85	8.25	8.92
FOXG_09028	NADP-dependent alcohol dehydrogenase VI	0.32	1.77	2.53	114.35
FOXG_09770	Transferase	0.09	0.77	21.01	90.05
FOXG_10443	Chitin synthase 2	0.01	0.29	0.99	2.47
FOXG_01655	Kinesin family protein	0.07	0.62	1.39	2.91
FOXG_06086	G2-specific protein kinase nim-1	0.06	0.45	1.25	1.31

Transcripts with defined functions were tabulated according to their FPKM values across four time points.
