# Supplementary material for: Genome wide transcriptome profiling of Fusarium oxysporum f sp. ciceris conidial germination reveals new insights into infection-related genes
Source: Sci Rep. 2016 Nov 17;6:37353. doi: 10.1038/srep37353 (PMC5112587; doi:10.1038/srep37353)
Supplement: Supplementary Information [file srep37353-s1.doc]

**Supplementary File**

**Genome wide transcriptome profiling of *Fusarium oxysporum* f sp. *ciceris* conidial germination reveals new insights into infection-related genes**

Mamta Sharma, Anindita Sengupta, Raju Ghosh, Gaurav Agarwal, Avijit Tarafdar, A Nagavardhini, Suresh Pande and Rajeev K Varshney

**Supplementary Figure 1.** **Global view of transcriptional changes by volcano plot using Kal’s statistical test (Z-test).** Log_2_ fold change in RPKM expression values (x-axis) versus -log10 (Bonferroni corrected P-values) (y-axis) were computed in volcano plots that arranges expressed genes along dimensions of biological as well as statistical significance. Figure A-F corresponding to *Foc*_0h vs *Foc*_2h, *Foc*_0h vs *Foc*_6h, *Foc*_0h vs *Foc*_24h, *Foc*_2h vs *Foc*_6h, *Foc*_2h vs *Foc*_24h and *Foc*_6h vs *Foc­_*24h respectively. The X-axis represents the log fold change on which blue dots on the left side of zero represents significant down regulated genes whereas blue dots on the right side of zero represents significant up-regulated genes. Y-axis represents the negative log of p-value of the performed statistical test where data points with low p-values (highly significant) appearing towards the top of the plot.


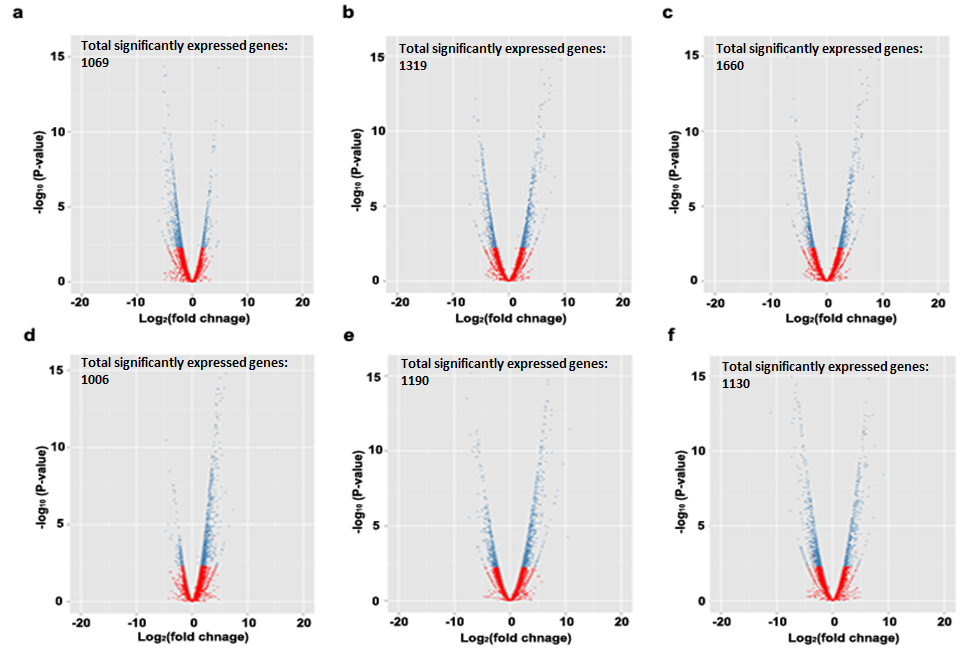


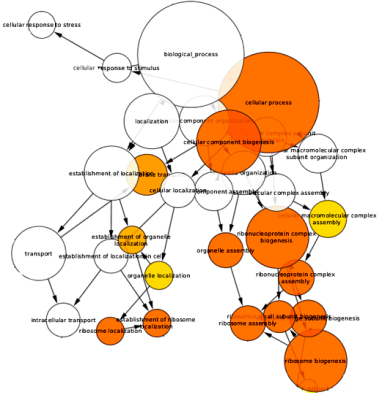
**Supplementary Figure 2.** **GO functional enrichment analysis of significantly down-regulated transcripts.**

**Supplementary Figure 3. Representation of the pentose and glucuronate interconversion pathway.** Up-regulated transcripts encoding enzymes identified at *Foc_*0h vs *Foc_*2h (E.C.1.1.1.10, 1.1.1.22) and *Foc_*0h vs *Foc_*24h (E.C.3.1.1.11) was annotated. Change in expression is indicated with heat map. (Please see the details of up-regulated transcripts involved in different pathways in Supplementary Table 4).


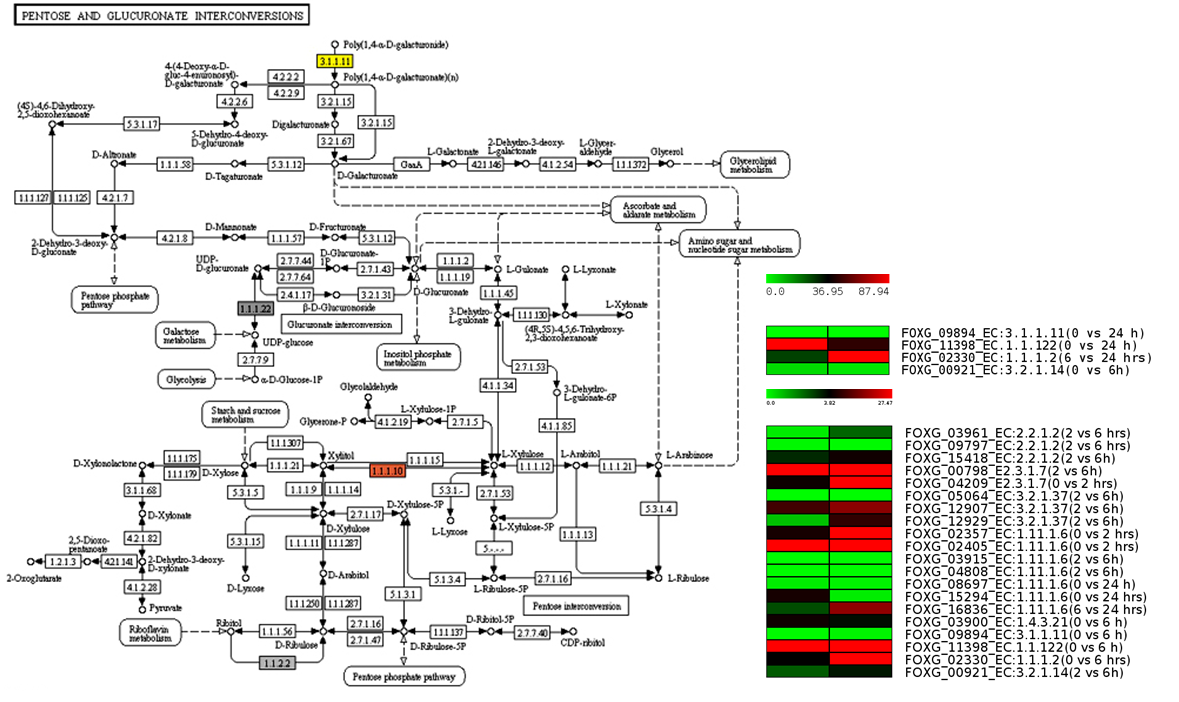


**Supplementary Figure 4.** **Representation of amino sugar and nucleotide sugar metabolism.** Up-regulated transcripts encoded enzymes identified at *Foc*_0h vs *Foc*_6h (E.C.2.4.1.16) and *Foc*_2h vs *Foc*_24h (E.C.3.2.1.14) was annotated.

**
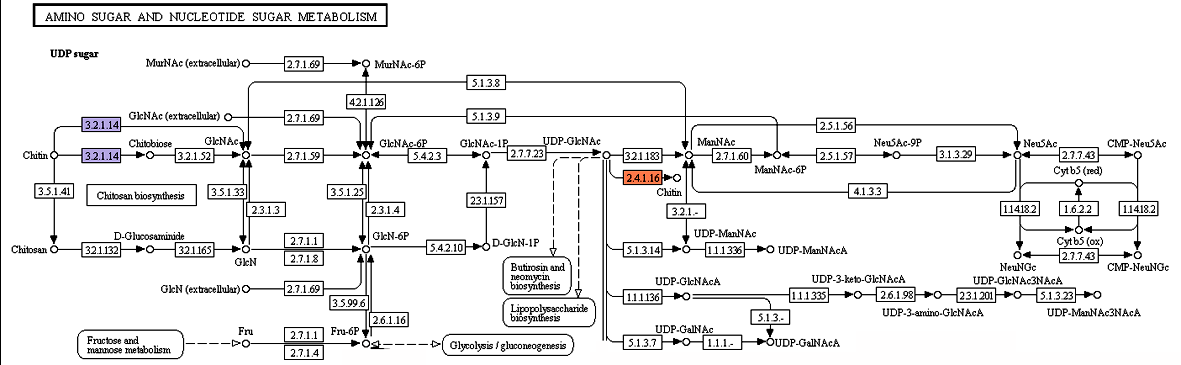
**

**Supplementary Figure 5.** **Representation of the amino acid metabolism pathway.** Gly-Ser-Thr pathway activated *Foc*_0h vs *Foc*_2h, *Foc*_0h vs *Foc*_6h, *Foc*_0h vs *Foc*_24h, *Foc*_2h vs *Foc*_24h.

**
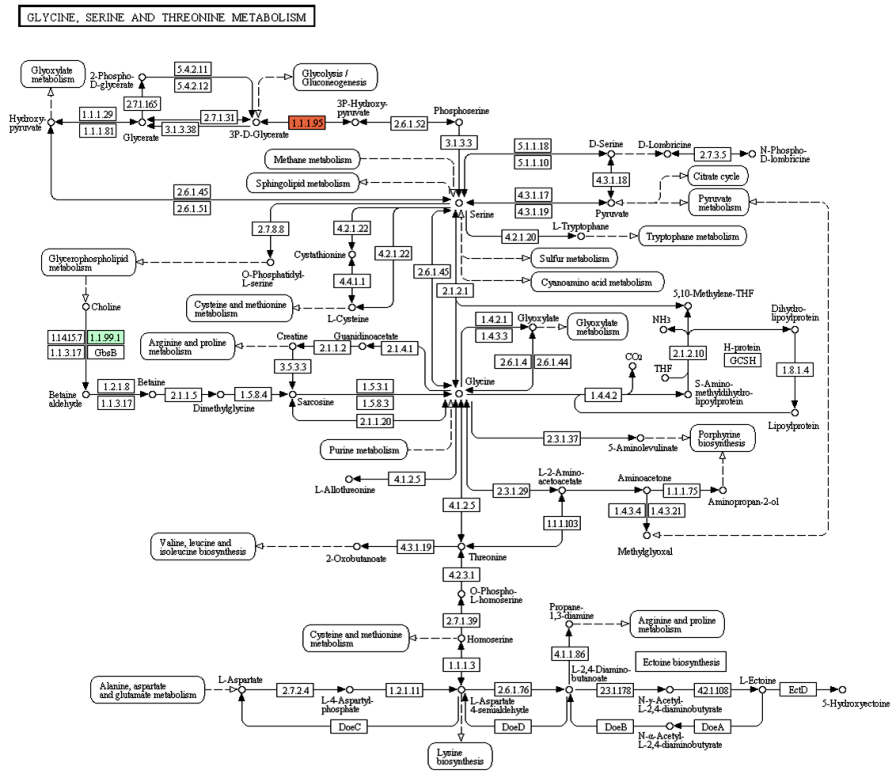
**

**Supplementary Figure 6. Representation of the bisphenol degradation pathway activated during *Foc*_0h vs *Foc*_6h.**

**
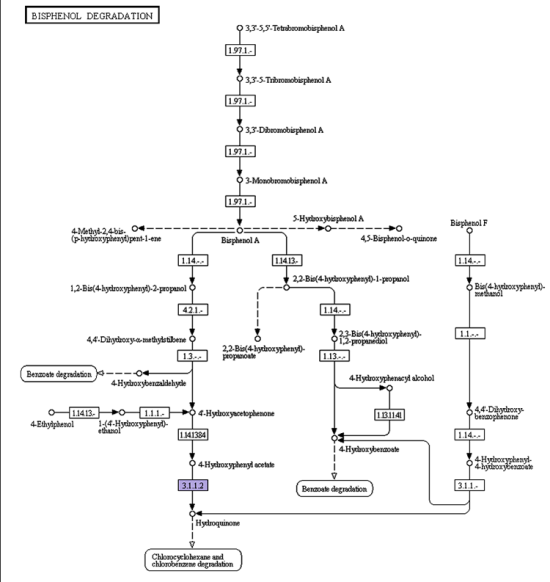
**

**Supplementary Figure 7.** **Representation of the isoquinoline alakaloid biosynthesis pathway activated during *Foc*_0h vs *Foc*_2h and *Foc*_0h vs *Foc*_6h. Change in expression is indicated with heat map.** (Please see the details of up-regulated transcripts involved in different pathways in Supplementary Table 4).

**
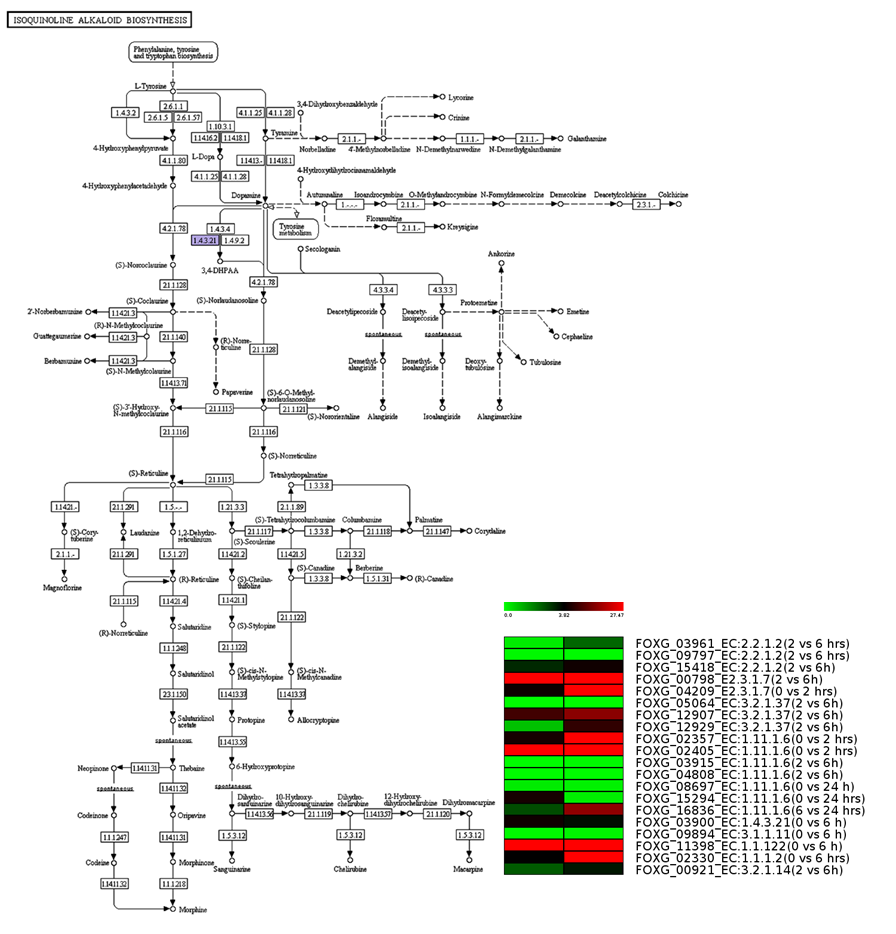
**

**Supplementary Table 1. Distribution of unique sequences of *Foc* assigned to GO analysis.**

**Up-regulated**

| **Molecular_function** | **828** |
| --- | --- |
| protein binding transcription factor activity | 5 |
| sequence-specific DNA binding transcription factor activity | 31 |
| catalytic activity | 512 |
| signal transducer activity | 8 |
| structural molecule activity | 50 |
| transporter activity | 100 |
| binding | 502 |
| electron carrier activity | 2 |
| potassium channel regulator activity | 1 |
| antioxidant activity | 5 |
| enzyme regulator activity | 8 |
| signaling receptor activity | 4 |
| translation regulator activity | 2 |
| **Cellular_component** | **754** |
| extracellular region | 43 |
| basement membrane | 1 |
| cell | 680 |
| cell-cell junction | 3 |
| plasmodesma | 1 |
| membrane | 210 |
| virion | 1 |
| extracellular matrix | 2 |
| membrane-enclosed lumen | 198 |
| macromolecular complex | 200 |
| mitochondrial nucleoid | 1 |
| organelle | 535 |
| extracellular region part | 8 |
| organelle part | 303 |
| membrane part | 185 |
| cell part | 680 |
| postsynaptic membrane | 2 |
| **Biological_process** | **890** |
| reproduction | 22 |
| immune system process | 8 |
| cell adhesion | 4 |
| metabolic process | 741 |
| cellular process | 702 |
| reproductive process | 11 |
| signaling | 22 |
| killing of cells of other organism | 2 |
| multicellular organismal process | 20 |
| developmental process | 25 |
| growth | 8 |
| locomotion | 4 |
| single-organism process | 486 |
| response to stimulus | 106 |
| localization | 166 |
| establishment of localization | 158 |
| multi-organism process | 22 |
| biological regulation | 146 |
| cellular component organization or biogenesis | 246 |

Down-regulated

| **Molecular_function** | **741** |
| --- | --- |
| transcription factor binding transcription factor activity | 4 |
| sequence-specific DNA binding transcription factor activity | 27 |
| catalytic activity | 539 |
| signal transducer activity | 11 |
| guanyl-nucleotide exchange factor activity | 3 |
| structural molecule activity | 4 |
| transporter activity | 73 |
| binding | 418 |
| electron carrier activity | 2 |
| antioxidant activity | 14 |
| metallochaperone activity | 1 |
| enzyme regulator activity | 19 |
| signaling receptor activity | 4 |
| translation regulator activity | 1 |
| nutrient reservoir activity | 1 |
|  |  |
| **Cellular_component** | **626** |
| extracellular region | 45 |
| cell | 565 |
| plasmodesma | 1 |
| membrane | 257 |
| virion | 2 |
| viral capsid | 1 |
| cell junction | 8 |
| membrane-enclosed lumen | 52 |
| macromolecular complex | 110 |
| mitochondrial nucleoid | 2 |
| organelle | 428 |
| extracellular region part | 8 |
| organelle part | 225 |
| membrane part | 209 |
| synapse part | 7 |
| cell part | 564 |
| synapse | 9 |
|  |  |
| **Biological_process** | **781** |
| reproduction | 31 |
| immune system process | 4 |
| cell adhesion | 2 |
| metabolic process | 623 |
| cellular process | 546 |
| reproductive process | 18 |
| signaling | 34 |
| multicellular organismal process | 32 |
| developmental process | 46 |
| growth | 8 |
| locomotion | 4 |
| single-organism process | 537 |
| response to stimulus | 149 |
| localization | 167 |
| establishment of localization | 156 |
| multi-organism process | 29 |
| biological regulation | 186 |
| cellular component organization or biogenesis | 108 |

**Supplementary Table 2: Differentially regulated transcripts taking into account in this study.** Genes associated with multiple biological process were categorized and tabulated according to their log_2_fold change value at six combinations of time points.

Carbohydrate metabolic process

| **Gene Id** | **Name** | **0h vs 2h** | **0h vs 6h** | **0h vs 24h** | **2h vs 6h** | **2h vs 24h** | **6h vs 24h** |
| --- | --- | --- | --- | --- | --- | --- | --- |
| FOXG_00556 | Hexokinase 1 | - | 2.57 | - | - | - | - |
| FOXG_00670 | Phosphoglucomutase | - | 4.59 | - | - | 3.34 | - |
| FOXG_03961 | Transaldolase | - | - | - | - | 3.03 | - |
| FOXG_04251 | Glycogenin | - | 2.72 | - | - |  | - |
| FOXG_12350 | Cytochrome P450 | - | 5.35 | - | - | 3.23 | - |
| FOXG_07494 | aldolase | - | - | - | 3.42 | - | - |
| FOXG_15445 | Pyruvate kinase | - | - | - | 2.05 | - | - |
| FOXG_06319 | Glycogen phosphorylase | - | 2.82 | - | 2.52 | - | - |
| FOXG_01733 | Pyruvate carboxylase | - | 2.77 | - |  | - | - |
| FOXG_13927 | 1,4-alpha-glucan branching enzyme | - | - | - | 2.14 | - | - |
| FOXG_03418 | Phosphoenolpyruvate carboxykinase | - | - | - | - | 2.45 | - |

**Nitrogen metabolism**

| **Gene Id** | **Name** | **0h vs 2h** | **0h vs 6h** | **0h vs 24h** | **2h vs 6h** | **2h vs 24h** | **6h vs 24h** |
| --- | --- | --- | --- | --- | --- | --- | --- |
| FOXG_04324 | Cysteine synthase B |  |  | - | - | 9.51 | 6 |
| FOXG_11520 | NAD-specific glutamate dehydrogenase | 2.37 | 3.61 | - | - | - | - |
| FOXG_01332 | N amino acid permease |  | 2.27 | - | - | - | - |
| FOXG_09346 | Ornithine aminotransferase | 2.66 | 3.29 | - | - | - | - |
| FOXG_09770 | Transferase | 3.16 | 7.93 | 10.03 | 4.78 | 6.88 | 2.1 |
| FOXG_12915 | Arginase | 3.27 | 5.14 | 3.97 | - | - | - |

**Cell communication**

| **Gene Id** | **Name** | **0h vs 2h** | **0h vs 6h** | **0h vs 24h** | **2h vs 6h** | **2h vs 24h** | **6h vs 24h** |
| --- | --- | --- | --- | --- | --- | --- | --- |
| FOXG_01655 | Kinesin family protein | 3.14 | 4.3 | 5.37 | - | - | - |
| FOXG_00162 | F-actin capping protein subunit beta | - | 3.22 | 2.46 | - | - | - |
| FOXG_00438 | Arp2/3 complex chain sop2 | - | 3.84 | 3.15 | - | - | - |
| FOXG_00407 | annexin XIV | - |  | 3 | - | - | - |
| FOXG_03179 | F-actin capping protein subunit alpha | - | 3.13 | 3.04 | - | - | - |
| FOXG_03650 | ARP2/3 complex 20 kDa subunit | - | 2.29 | 2.95 | - | - | - |
| FOXG_11105 | ARP2/3 complex 21 kDa subunit | - | 2.82 | - | - | - | - |
| FOXG_05467 | Actin cortical patch component | - | 2.51 | - | 2.22 | - | - |
| FOXG_00070 | Actin binding protein | - | 3.17 | - | - | - | - |
| FOXG_02102 | Actin-binding protein | - | 3.04 | 2.53 | - | - | - |
| FOXG_13729 | Actin-85C | - | 2.66 | 2.66 | - | - | - |

Cellular transporters

| **Gene Id** | **Name** | **0h vs 2h** | **0h vs 6h** | **0h vs 24h** | **2h vs 6h** | **2h vs 24h** | **6h vs 24h** |
| --- | --- | --- | --- | --- | --- | --- | --- |
| FOXG_05915 | Plasma membrane zinc ion transporter | - | - | 3.39 | - | 4.53 | 5.28 |
| FOXG_11983 | ABC transporter | - | 3.04 | 3.32 | 2.86 | 3.14 |  |
| FOXG_04325 | MFS drug transporter | - | - | - | - | 8.53 | 7.59 |
| FOXG_09760 | mfs-multidrug-resistance transporter | 2.11 | 2.69 | - | - | - | - |
| FOXG_01635 | ABC transporter | - | 2.55 | - | - | - | - |
| FOXG_03983 | Zinc-regulated transporter 1 | - | 3.33 | 5.43 | 3.6 | 5.7 | - |
| FOXG_08139 | Protein transporter Sec24 | - | 2.96 | - | - | - | - |
| FOXG_11327 | ABC transporter Adp1 | - | 2.73 | - | - | - | - |
| FOXG_15712 | ABC transporter | - | - | 3.08 | - | - | - |
| FOXG_03843 | Siderochrome-iron transporter | - | - | 5.26 | - | 5.58 | - |
| FOXG_07770 | Copper ion transmembrane transport | - | - | 2.6 | - |  | - |
| FOXG_07498 | MSF transporter | - | - | 6.12 | - | 5.72 | - |
| FOXG_08743 | Cation transport | - | - | 2.76 | - |  | - |
| FOXG_11427 | Sugar transporter | - | - | 2.95 | - | 2.66 | - |
| FOXG_12291 | Urea transporter Dur3 | - | - | - | 6.14 |  | - |
| FOXG_12338 | Amino-acid permease inda1 | - | - | - | 2.12 |  | - |
| FOXG_13371 | Siderophore iron transporter mirB | - | - | - | 3.58 | 4.34 | - |
| FOXG_04747 | Transporter protein SMF1/ESP1 | - | - | - | 2.92 |  | - |
| FOXG_17607 | Maltose porter | - | - | - | 3.09 | 4.55 | - |
| FOXG_07388 | Nucleoside transporter | - | - | - | 3.65 |  | - |
| FOXG_00527 | Ammonium transporter MEP1 | - | - | - | 2.13 |  | - |
| FOXG_08943 | Trehalose transporter | - | - | - | 2.88 |  | - |
| FOXG_04577 | ABC transporter | - | - | - | - | 2.98 | - |
| FOXG_07525 | Siderochrome-iron transporter | - | - | - | - | 3.17 | 2.79 |
| FOXG_00347 | Sucrose transporter | - | - | - | - | 2.37 |  |
| FOXG_09782 | Siderophore iron transporter mirB | - | - | - | - | 3.4 | 2.3 |
| FOXG_11984 | MSF membrane transporter | - | - | - | - | 2.26 |  |
| FOXG_15714 | Siderophore iron transporter mirA | - | - | - | - | 2.65 | 3.18 |
| FOXG_03843 | Siderochrome-iron transporter | - | - | - | - | 5.58 | 4.66 |
| FOXG_07498 | MSF transporter | - | - | - | - | - | 4.21 |
| FOXG_09887 | Major facilitator superfamily transporter protein superfamily | - | - | - | - | - | 3.06 |
| FOXG_15712 | ABC transporter | - | - | - | - | - | 2.71 |

Cell wall biosynthesis and membrane modification

| **Gene Id** | **Name** | **0h vs 2h** | **0h vs 6h** | **0h vs 24h** | **2h vs 6h** | **2h vs 24h** | **6h vs 24h** |
| --- | --- | --- | --- | --- | --- | --- | --- |
| FOXG_01953 | Extracellular cell wall glucanase Crf1 | - | - | 3.16 | - | 2.72 | - |
| FOXG_11947 | Extracellular cell wall glucanase Crf1 | - | - | - | - | 2.77 | - |
| FOXG_10638 | Endoglucanase-5 |  | 3.87 | 2.72 | 3.36 | 2.21 | - |
| FOXG_17723 | Cell wall macromolecule catabolic process |  |  | 6.39 | - | 4.99 | 4.64 |
| FOXG_08810 | Cell wall glucanosyltransferase | 2.41 | 3.66 |  | - | - | - |
| FOXG_12882 | Endochitinase 1 precursor | - | - | 2.71 | - | 2.3 | - |
| FOXG_03822 | Cell wall macromolecule catabolic process | - | - | - | - | 3.17 | 2.21 |
| FOXG_12330 | Pectin methylesterase | - | - | 6 | - | - | 4.5 |
| FOXG_10443 | Chitin synthase 2 | 4.65 | 6.43 | 7.74 | - | 3.09 | - |

Peroxisome biogenesis

| **Gene Id** | **Name** | **0h vs 2h** | **0h vs 6h** | **0h vs 24h** | **2h vs 6h** | **2h vs 24h** | **6h vs 24h** |
| --- | --- | --- | --- | --- | --- | --- | --- |
| FOXG_08573 | Peroxisome biogenesis factor 7 | 2.56 | 3.56 | - | - | - | - |

**Fatty acid metabolism**

| **Gene Id** | **Name** | **0h vs 2h** | **0h vs 6h** | **0h vs 24h** | **2h vs 6h** | **2h vs 24h** | **6h vs 24h** |
| --- | --- | --- | --- | --- | --- | --- | --- |
| FOXG_02375 | Acetyl-CoA carboxylase | - | 2.68 | - | 4.33 | 3.27 | - |
| FOXG_08347 | Acyl-CoA-binding protein | - | 3.23 | - | - | - | - |
| FOXG_13905 | Long-chain-fatty-acid-CoA ligase | - | - | - | 3.1 | 7.43 | - |
| FOXG_06392 | Fatty acid synthase subunit beta dehydratase | - | - | - | 3.5 | 2.68 | - |
| FOXG_06391 | Fatty acid synthase subunit alpha reductase | - | - | - | 3.29 | 2.29 | - |
| FOXG_08523 | Acyl-CoA desaturase | - | - | - | 3.4 | 3.64 | - |
| FOXG_08835 | Delta-12fatty acid desaturase | - | - | - | 3.65 | 2.64 | - |
| FOXG_02011 | 3-hydroxybutyryl-CoA dehydrogenase | - | - | - | 2.12 |  | - |
| FOXG_14342 | Fatty acid synthase subunit beta dehydratase | - | - | - | 3.29 | 3.25 | - |
| FOXG_09784 | Enoyl-CoA hydratase | - | - | - | - | 3.92 | 2.03 |
| FOXG_02126 | Enoyl-CoA hydratase, mitochondrial precursor | - | 2.85 | 2.79 |  | - | - |
| FOXG_15688 | Enoyl-CoA hydratase/isomerase | - |  | - | 3.41 | - | - |
| FOXG_12929 | Enoyl Coenzyme A hydratase domain containing 3 | - | 4.17 | - | 3.19 | - | - |
| FOXG_04209 | Carnitine acetyl transferase | 2.41 | 2.84 | - | - | - | - |
| FOXG_06336 | Mitochondrial carnitine:acyl carnitine carrier | - | 2.3 | - | - | - | - |
| FOXG_16836 | Peroxisomal catalase | - | - | - | - |  | 2.69 |
| FOXG_02405 | Catalase 1 | - | - | 3.33 | - |  | 2.75 |
| FOXG_12260 | Peroxidase/catalase 2 |  | - |  | 2.69 | 2.7 | - |
| FOXG_02357 | Catalase | 2.51 | - | 4.05 |  |  | - |
| FOXG_04389 | Superoxide dismutase, mitochondrial precursor | - | - | 6.07 | 3.36 | 5.49 | - |
|  |  |  | - |  |  |  |  |

Apoptosis inducing factor

| **Gene Id** | **Name** | **0h vs 2h** | **0h vs 6h** | **0h vs 24h** | **2h vs 6h** | **2h vs 24h** | **6h vs 24h** |
| --- | --- | --- | --- | --- | --- | --- | --- |
| FOXG_15750 | Apoptosis inducing factor | - | 2.26 | 5.35 | 3.24 | 6.34 | 3.09 |
| FOXG_06044 | Metacaspase-1 precursor | - | 3.26 | 3.02 | 2.22 | - | - |

Ribosome biogenesis, proteasome components, and autophagy

| **Gene Id** | **Name** | **0h vs 2h** | **0h vs 6h** | **0h vs 24h** | **2h vs 6h** | **2h vs 24h** | **6h vs 24h** |
| --- | --- | --- | --- | --- | --- | --- | --- |
| FOXG_10614 | Ubiquitin C-terminal hydrolase | 2.05 | - | - | - | - | - |
| FOXG_07470 | Ubiquitin C-terminal hydrolase L3 | - | 4.15 | - | - | - | - |
| FOXG_07962 | Proteasome subunit alpha type 1 | - | 2.72 | - | - | - | - |
| FOXG_00173 | npl4 protein | - | 2.89 | 2.51 | - | - | - |
| FOXG_00266 | Multiubiquitin chain binding protein | - | 2.77 | - | - | - | - |
| FOXG_00491 | Proteasome component C1 | - | 2.75 | - | - | - | - |
| FOXG_08381 | Ubiquitin-activating enzyme E1 1 | - | 2.43 | - | - | - | - |
| FOXG_08551 | Proteasome component PUP2 | - | 2.95 | - | - | - | - |
| FOXG_10614 | Ubiquitin C-terminal hydrolase | - | 4.26 | - | 2.2 | - | - |
| FOXG_11622 | Ubiquitin conjugating enzyme UbcB | - | 2.4 | - | - | - | - |
| FOXG_02134 | Proteasome subunit alpha type 7 | - | 2.32 | - | - | - | - |
| FOXG_13824 | Proteasome component Y7 | - | 2.95 | - | 2.17 | - | - |
| FOXG_15663 | Polyubiquitin binding protein Doa1/Ufd3 | - | 2.66 | 2.86 | - | - | - |
| FOXG_03651 | Ubiquitin carboxyl-terminal hydrolase 6 | - | 2.4 | - | - | - | - |
| FOXG_05173 | Proteasome subunit alpha type 6 | - | 2.51 | - | 2.02 | - | - |
| FOXG_07470 | Ubiquitin C-terminal hydrolase L3 | - | 4.15 | - | 4.24 | - | - |
| FOXG_07962 | Proteasome subunit alpha type 1 | - | 2.72 | - | 2.03 | - | - |
| FOXG_03134 | Ubiquitin carboxyl-terminal hydrolase | - |  | - | 3.62 | - | - |

**Genes involved in protein kinase activity**

| **Gene Id** | **Name** | **0h vs 2h** | **0h vs 6h** | **0h vs 24h** | **2h vs 6h** | **2h vs 24h** | **6h vs 24h** |
| --- | --- | --- | --- | --- | --- | --- | --- |
| FOXG_06086 | G2-specific protein kinase nim-1 | 2.79 | 4.28 | 4.35 | - | - | - |
| FOXG_01680 | serine/threonine-protein kinase chk2 |  | 2.41 | - | - | - | - |
| FOXG_01946 | Calcium/calmodulin-dependent protein kinase | - | 2.77 | - | 2.08 | - | - |
| FOXG_05517 | cAMP-dependent protein kinase regulatory subunit | - | 4 | 3.66 | 2.2 | - | - |
| FOXG_10436 | Uridine kinase | - | 3.07 | 3.09 | - | - | - |
| FOXG_03853 | Protein kinase domain-containing protein | - | - | 3.91 | - | 4.52 | 2.78 |
| FOXG_08525 | Protein kinase domain-containing protein | - | - | 2.98 | - | 3.76 | 2.23 |
| FOXG_08759 | Serine/threonine-protein kinase 24 | - | - | 2.94 | - | 2.67 | 2.51 |
| FOXG_11284 | Ribitol kinase | - | - | - | 2.51 | - | - |
| FOXG_05092 | Mitogen-activated protein kinase spm1 | - | - | - | 2.39 | - | - |

Stress response

| **Gene Id** | **Name** | **0h vs 2h** | **0h vs 6h** | **0h vs 24h** | **2h vs 6h** | **2h vs 24h** | **6h vs 24h** |
| --- | --- | --- | --- | --- | --- | --- | --- |
| FOXG_11750 | Protein rds1 | 2.49 | - | 6.56 | - | 4.07 | 5.82 |

Genes involved in conidiation specific protein

| **Gene Id** | **Name** | **0h vs 2h** | **0h vs 6h** | **0h vs 24h** | **2h vs 6h** | **2h vs 24h** | **6h vs 24h** |
| --- | --- | --- | --- | --- | --- | --- | --- |
| FOXG_13755 | Clock-controlled-9 protein | - | - | 3.93 | 2.7 | 5.35 | 2.65 |
| FOXG_12143 | Phytoene dehydrogenase | - | - | 7.92 | - | 6.39 | 5.47 |
| FOXG_15406 | Opsin-1 | - | - | 3.76 | - | 2.86 | 3.69 |
| FOXG_06342 | Protein bli-3 | - | - | 4.02 | - | 2.36 | 2.64 |
| FOXG_01269 | Conidiation-specific protein 10 | - | - | - | - | - | 5.27 |

**Down regulated transcripts**

Ribosome biogenesis

| **Gene Id** | **Name** | **0h vs 2h** | **0h vs 6h** | **0h vs 24h** | **2h vs 6h** | **2h vs 24h** | **6h vs 24h** |
| --- | --- | --- | --- | --- | --- | --- | --- |
| FOXG_01019 | Ribosome biogenesis protein Pescadillo | -3.27 | -4.84 | -5.44 | - | - | - |
| FOXG_00216 | Ribosome biogenesis protein RPF2 | -2.76 | -3.96 | -3.76 | - | - | - |
| FOXG_08506 | Ribosomal RNA assembly protein mis3 | -2.79 | -3.71 | -4.24 | - | - | - |
| FOXG_08265 | Ribosome biogenesis protein Bms1 | -3.03 | -4.06 | -4.37 | - | - | - |
| FOXG_09435 | Ribosome production factor 1 | -3.28 | -4.44 | -5.24 | - | - | - |
| FOXG_10250 | Eukaryotic ribosome biogenesis protein 1 | -2.71 | -4.38 | -4.92 | - | - | - |
| FOXG_10350 | Ribosome biogenesis protein Rrb1 | -2.46 | -2.77 | -3.39 | - | - | - |
| FOXG_01853 | 60S ribosome subunit biogenesis protein NIP7 | -2.33 | -3.86 | -4.17 | - | - | - |
| FOXG_02230 | 60S acidic ribosomal protein | -2.89 | -4.69 | -5.93 | - | - | - |
| FOXG_01695 | Regulator of ribosome biosynthesis | -2.55 | -4.07 | -4.54 | - | - | - |
| FOXG_02212 | Ribosome biogenesis protein RLP24 | -2.23 | -3.31 | -4.29 | - | - | - |
| FOXG_13912 | Ribosome biogenesis protein BRX1 | -2.61 | -4.15 | -4.68 | - | - | - |
| FOXG_00371 | 60S ribosomal protein L35 | - | - | -3 | - | -2.92 | - |
| FOXG_00802 | 60S ribosomal protein L15 | - | - | -2.98 | - | -2.92 | -2.59 |
| FOXG_09282 | 60S acidic ribosomal protein P2 | - | - | -3.09 | - | -3.62 | - |
| FOXG_11195 | Ribosomal L18ae protein family | - | - | -3.12 | - | - | - |
| FOXG_01896 | 60S ribosomal protein L10a | - | - | -3.33 | - | - | -2.97 |
| FOXG_02122 | 40S ribosomal protein S22 | - | - | -2.84 | - | - | -2.17 |
| FOXG_01998 | 60S acidic ribosomal protein P0 | - | - | -2.89 | - | - | -2.78 |
| FOXG_03277 | 60S ribosomal protein L8 | - | - | -3.14 | - | - | - |
| FOXG_03375 | 60S ribosomal protein L14-B | - | - | -2.93 | - | - | - |
| FOXG_04217 | 60S ribosomal protein L12 | - | - | -3.88 | - | - | - |
| FOXG_05325 | 60S ribosomal protein L22 | - | - | -3.11 | - | - | - |
| FOXG_05398 | 60S ribosomal protein L5 | - | - | -3.33 | - | - | - |
| FOXG_07590 | 60S ribosomal protein L38 | - | - | -2.63 | - | - | - |
| FOXG_09399 | 40S ribosomal protein S20 | - | - |  | - | -2.84 | - |
| FOXG_09465 | 40S ribosomal protein S3 | - | - |  | - | -2.86 | - |

**Supplementary Table 3. Table S5**. Differentially expressed transcripts selected for pathway analysis at six combinations of time points.

| **Time points** | **Up-regulated** | **Down-regulated** |
| --- | --- | --- |
| *Foc*_0h vs *Foc*_2h | 42 | 60 |
| *Foc*_0h vs *Foc*_6h | 68 | 62 |
| *Foc*_0h vs *Foc*_24h | 61 | 67 |
| *Foc*_2h vs *Foc*_6h | 56 | 31 |
| *Foc*_2h vs *Foc*_24h | 61 | 58 |
| *Foc*_6h vs *Foc*_24h | 46 | 49 |

**Supplementary Table 4. Major pathways up-regulated at different combination of time points.**

| **Time points** | **EC number** | **FOXG Number** | **Pathways activated** | **Enzyme name** |
| --- | --- | --- | --- | --- |
| ***Foc*_0h vs *Foc*_2h** | EC 1.1.1.22, EC 1.1.1.10 | FOXG_11398, FOXG_11607 | Pentose and glucorunate interconversion | UDP-glu-6-dehydrogenase, 3-oxo-acyl-reductase-ACP reductase |
|  | EC 1.2.1.16 | FOXG_00813 | Ala, Asp and glutamate metabolism | Succinate semialdehyde dehydrogenase |
|  | EC 1.4.3.21 | FOXG_03900 | Gly, Ser and Thr metabolism | Primary amine oxidase |
|  | EC 3.5.3.1 | FOXG_11179 | Arg and pro metabolism | Arginase |
|  | EC 2.6.1.19 | FOXG_02078 | B-Ala metabolism | 4-aminobutyrate aminotransferase |
|  | EC 2.4.1.16 | FOXG_00113 | Amino sugar and nucleotide sugar metabolism | Chitine synthase |
| ***Foc*_0h vs *Foc*_6h** | EC 3.5.3.1 | FOXG_11179 | Arg and pro metabolism | Arginase |
|  | EC 1.4.3.21 | FOXG_03900 | Tyr metabolism | Primary amine oxidase |
|  | EC 2.4.1.16 | FOXG_00113 | Amino sugar and nucleotide sugar metabolism | Chitine synthase |
| ***Foc*_0h vs *Foc*_24h** | EC 1.1.1.2 | FOXG_02330 | Glycolysis and gluconeogenesis | Alcohol dehydrogenase |
|  | EC 1.1.1.95 | FOXG_01425 | Gly, Ser and Thr metabolism | 3-phosphoglycerate dehydrogenase |
|  | EC 3.1.1.11 | FOXG_09894 | Starch and sucrose metabolism | Pectinesterase |
|  | EC 3.2.1.14, EC 2.4.1.16 | FOXG_00921, FOXG_00113 | Amino sugar and nucleotide sugar metabolism | Chitinase, Chitine synthase |
|  | EC 4.1.1.65 | FOXG_04268 | Glycerophospholipid metabolism | Phosphatidyl serine decarboxylase |
|  | EC 3.1.1.47 | FOXG_07636 | Ether lipid metabolism | Platelet activating factor |
| ***Foc*_2h vs *Foc*_6h** | EC 1.14.13.8 | FOXG_01805 | Drug metabolism | Dimethyl aniline monooxygenase 2 |
| ***Foc*_2h vs *Foc*_24h** | EC 1.1.1.2 | FOXG_02330 | Glycolysis and gluconeogenesis | Alcohol dehydrogenase |
|  | EC 5.4.2.8 | FOXG_01689 | Fructose and mannose metabolism | Phosphomannomutase |
|  | EC 1.1.99.1 | FOXG_08873 | Gly, Ser and Thr metabolism | Choline dehydrogenase |
|  | EC 3.2.1.14 | FOXG_00921 | Amino sugar and nucleotide sugar metabolism | Chitinase |
|  | EC 1.1.1.95 | FOXG_01425 | Methane metabolism | D-3-Phosphoglycerate dehydrogenase |
|  | EC 1.14.13.8 | FOXG_01805 | Drug metabolism | Dimethyl aniline monooxygenase 2 |
| ***Foc*_6h vs *Foc*_24h** | EC 1.1.1.2 | FOXG_02330 | Caprolactum degradation | Alcohol dehydrogenase |

Supplementary Table 5. The list and expression profiles of selected differentially expressed transcripts validated with qPCR analysis.

| **Gene Id** | **Name** | **Primer’s name** | **(5'→3') Primer sequence (F/R)** | **Expression profile*** |
| --- | --- | --- | --- | --- |
| FOXG_06319 | Glycogen phosphorylase | Gly-Phos | CAAGGACCTCACCACTCTAAAC/ CGCTTGTACTCGTGGATTCT | 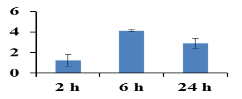 |
| FOXG_01733 | Pyruvate carboxylase | Pyru_carbo | CTCCAACTTGTACTCCCTTGAG/ GAACCAGCTTTCGCATCTTTC | 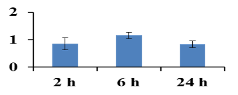 |
| FOXG_12915 | Arginase | Argin | AGCGACAGCGGAAACATT/ CCATGCTCACGGAGAATCTT | 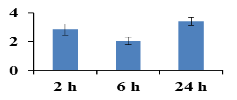 |
| FOXG_09784 | Enoyl coA hydratase | Enoyl-CoA | GAGCGGAAAGAAACCCATTATC/ TCTTGGTAGAGAACCTGCATTAG | 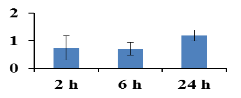 |
| FOXG_03099 | Malate synthase | Mal-Syn | CGACTTCAAGCAGGGACAAA/ TCGAAGAGGGAACCAGAGATAG | 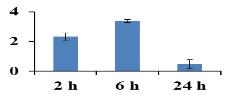 |
| FOXG_05915 | Zinc-regulated transporter | Zn-trans | CCCACTGCTTTCATCTCTCTC/ TGGGTGAAAGACCATCTCAATAA | 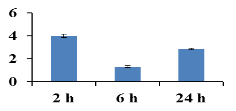 |
| FOXG_04325 | MFS transporter | MFS-trans | GCCACCAAATCCGGAGTAAT/ TTTCCCAACGCCCGTATT | 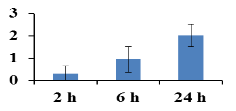 |
| FOXG_11983 | ABC transporter | ABC-trans | CTGGCAGACCGTTACATACTT/ TGAAGAACGTGCATGGGATAAG | 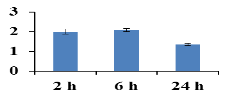 |
| FOXG_01953 | Glucanase Crf1 | Glucan-crf_1 | CTTCTCCAAAGGCGAGGTAAA/ GAACGAGCGACGATACGATAC | 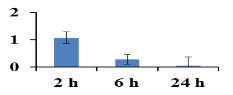 |
| FOXG_11947 | Glucanase Crf1 | Glucan-crf_2 | GGAGTGGCTTGGAGGTAAAG/ CCTTGGTCCAGTGAATGGTATAG | 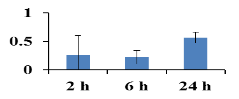 |
| FOXG_10638 | Endoglucanase-5 | Endoglucan | GAAGGTCCGTGGAACCAAA/ TGACACACTTGCTTCCAGTAG | 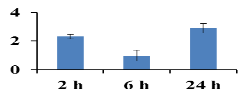 |
| FOXG_17723 | Cell wall macromolecule catabolic process | Macromole_1 | CCTTCTGGACCTGGACAATATC/ GATGGTACTGGTCTTGCTGTG | 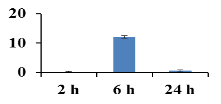 |
| FOXG_08810 | Glucanosyltransferase | Gluca-trans | GCAGATCAAGCTCGGAACA/ CGACAATGGAGATGCTCTTGTA | 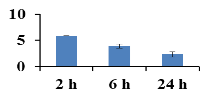 |
| FOXG_12882 | Endochitinase | Endochitin | AGACTTTGAGCCAGCCTTATC/ GTCCCAAGACCCAGCATAAT | 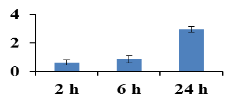 |
| FOXG_03822 | Cell wall macromolecule catabolic process | Macromole_2 | CTCGGCTACAAGGGTTCTTC/ CACACAGATGCGATCGTTAATC | 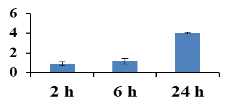 |
| FOXG_12330 | Pectin methyl esterase | Pectin-methyl | ACGGATACACTACCGACACTA/ GGCAACATTGACGTTGTAAAG | 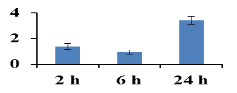 |
| FOXG_13795 | Pectin esterase | Pectin-ester | GCTCCACAACTGTCTACACTAC/ GATGGTGGTTCCGACATCAA | 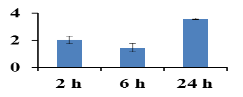 |
| FOXG_13755 | Clock controlled-9 protein | Clock-cont-9 | CGCCAATGACCAGCTTCT/ GAGCCTCAGAGACCTTGATTTC | 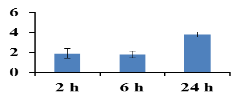 |
| FOXG_12143 | Phytoene dehydrogenase | Phyto-dehy | GAACTCAACGCTCACAACATTT/ TGACCAACAGGGACAAGAAC | 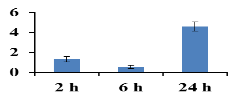 |
| FOXG_15406 | Opsin-1 protein | Opsin-1 | TGTACCACCGTGAGAGATCA/ CAATGACCGCAAAGATGGTATG | 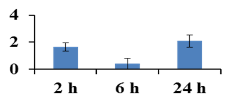 |
| FOXG_06342 | Protein bli-3 | Bli-3 | GTAACCTTGTATCTCGCTGCAT/ CGTGTTGGTGTGGAAGAGAA | 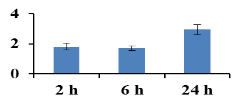 |
| FOXG_05517 | c-AMP dependent protein kinase regulatory protein | cAMP | GAGGTCATCATCAACGAAGGAG/ GAGACGTGGCAACGATACTA | 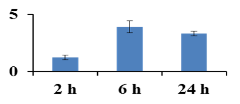 |
| FOXG_08525 | Protein kinase domain containing protein | Prot_kinase_1 | CGAGGTGTGGATTTAGGAGATG/ GTGCTGATTCGTTGTACGTTATTC | 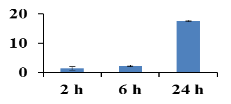 |
| FOXG_03853 | Protein kinase domain containing protein | Prot_kinase_2 | GGCCAGGTCCAATCCTATTATC/ CGCTCCAGATATCCACAGAATG | **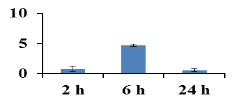** |
| - | 18S reference gene | 18S | CGCCAGAGGACCCCTAAAC/ ATCGATGCCAGAACCAAGAGA | **-** |

*X and Y axis denote the time points and corresponding Ct values, respectively.
